# Enhanced antitumor activity of combined hepatic arterial infusion chemotherapy with Lenvatinib and PD-1 inhibitors in unresectable hepatocellular carcinoma: a meta-analysis

**DOI:** 10.3389/fonc.2025.1513394

**Published:** 2025-02-12

**Authors:** Lingling Zhao, Cheng Xu, Jiewen Deng, Yang Ni

**Affiliations:** ^1^ Department of General Surgery, Sichuan Science City Hospital, Mianyang, China; ^2^ Department of Hepatobiliary Surgery, Affiliated Tumor Hospital of Guangxi Medical University, Nanning, China

**Keywords:** hepatic arterial infusion chemotherapy (HAIC), hepatocellular carcinoma (HCC), Lenvatinib, PD-1 inhibitors, meta-analysis, unresectable cancer

## Abstract

**Background:**

Hepatic arterial infusion chemotherapy (HAIC) is increasingly recognized as a primary treatment option for patients with unresectable hepatocellular carcinoma (uHCC), providing a focused treatment for localized tumors. The combination of lenvatinib, a multikinase inhibitor, with PD-1 inhibitors has demonstrated significant survival benefits in HCC. This meta-analysis aims to assess whether the integration of HAIC with lenvatinib and PD-1 inhibitors (referred to as the HAIC-L-P group) leads to better treatment effectiveness and security compared to lenvatinib and PD-1 inhibitors alone (L-P group) in uHCC.

**Methods:**

An exhaustive search of the literature was conducted, including PubMed, the Cochrane Library, Embase, ClinicalTrials.gov, and Web of Science, from the start of each database until September 2024, to ensure a thorough and up-to-date compilation of relevant studies. Extract data on outcome measures such as overall survival (OS), progression-free survival (PFS), objective response rate (ORR), disease control rate (DCR), and adverse events (AEs). Subsequently, meta-analyses were performed using RevMan 5.4 to quantitatively evaluate the aggregated effect of the HAIC-L-P regimen versus the L-P regimen alone.

**Results:**

In our systematic meta-analysis of eight retrospective cohort studies, the HAIC-L-P regimen demonstrated markedly enhanced OS, with an HR of 0.54 (95% CI: 0.45-0.64; p < 0.00001), and enhanced 1-year and 2-year OS rates. Superior PFS was also observed in the HAIC-L-P group, with an HR of 0.64 (95% CI: 0.55-0.75; p < 0.0001), and higher 1-year and 2-year PFS rates. Response rates were markedly higher in the HAIC-L-P group, with an ORR risk ratio of 2.15 (95% CI: 1.84-2.50; p < 0.00001) and a DCR risk ratio of 1.28 (95% CI: 1.20-1.43; p < 0.0001). The AEs classified as grade 3 or above were elevated in the HAIC-L-P group, with notable risk ratios for vomiting, elevated AST, elevated ALT, thrombocytopenia, neutropenia, and hyperbilirubinemia. No life-threatening AEs were reported.

**Conclusion:**

The HAIC-L-P regimen correlated with enhanced tumor responses and prolonged survival, alongside manageable adverse effects, indicating its potential as a viable therapeutic strategy for individuals afflicted with uHCC.

**Systematic review registration:**

https://www.crd.york.ac.uk/PROSPERO/, identifier CRD42024594109.

## Introduction

Hepatocellular carcinoma (HCC) is a prevalent malignancy that accounts for a significant proportion of cancer-related deaths globally (1). For early-stage HCC that is localized within the liver, surgical intervention or ablation remains the treatment of choice (2). Unfortunately, a substantial number of victims present with advanced-stage disease have progressed beyond the scope of resectability. Consequently, there has been a surge of interest in combined therapeutic strategies for unresectable hepatocellular carcinoma (uHCC). Systemic therapy opens new treatment possibilities for uHCC patients, significantly improving patient outcomes (3). For individuals with advanced HCC, lenvatinib, a multi-pathway tyrosine kinase inhibitor, is recommended for systemic therapy, offering satisfactory survival durations and therapeutic efficacy (4, 5). Immunotherapeutic agents, such as PD-1/L1 inhibitors, bolster T-cell activity and exert significant antitumor effects. These agents have demonstrated encouraging therapeutic results for individuals grappling with advanced HCC (6). Beyond systemic therapies, local treatment modalities have demonstrated the capacity to enhance treatment outcomes in patients battling advanced HCC. Hepatic arterial infusion chemotherapy (HAIC) entails the ongoing delivery of chemotherapy drugs via a catheter placed in the hepatic artery, increasing local intrahepatic drug concentrations while reducing systemic toxicity (7). Research has suggested that HAIC is capable of substantially extending the overall survival (OS) for individuals suffering from advanced HCC, and emerges as a viable and secure therapeutic strategy for those with portal vein invasion (8, 9).

A range of combined therapeutic strategies have demonstrated the potential to enhance patient outcomes and possibly transform uHCC into a resectable condition. Considering the unique anti-cancer properties of tyrosine kinase inhibitors (TKIs), PD-1 inhibitors, and hepatic arterial infusion chemotherapy (HAIC), the concurrent use of these three treatment modalities could lead to complementary advantages and show significant potential for securing positive treatment results in advanced HCC patients. In recent clinical investigations, the integration of HAIC with lenvatinib and PD-1 inhibitors has exhibited encouraging outcomes (10, 11). Consequently, our systematic meta-analysis was designed to assess the comparative effectiveness and safety of this three-pronged approach versus the dual regimen of PD-1 inhibitors linked with lenvatinib, to identify more potent therapeutic strategies for uHCC sufferers.

## Materials and methods

Adhering to the PRISMA guidelines (12), the article presents outcomes with rigorous transparency and detail. The meta-analysis has been registered with PROSPERO(CRD42024594109).

### Search strategy

Our literature retrieval strategy encompassed a systematic search of English-language databases such as PubMed, EMBASE, ClinicalTrials.gov, the Cochrane Library, and the Web of Science. The search terms we utilized spanned a range of relevant medical subject headings (MeSH terms), encompassing hepatocellular carcinoma’s various designations like “liver cancer” or “HCC”, as well as terms specific to our treatment of interest, including “hepatic arterial infusion chemotherapy” or “HAIC”, and the immunotherapeutic agents “PD-1/L1” and “ immunological checkpoint inhibitors”. Additionally, we incorporated the targeted therapy drug “Lenvatinib” or its trade name “Lenvima” into our search criteria to ensure comprehensive coverage of the topic. The search language is limited to English, and the search continues until September 30, 2024. Two researchers independently searched according to the unified search strategy and the literature was rigorously evaluated and selected based solely on the established criteria for inclusion and exclusion.

### Study selection

Inclusion criteria: 1) study design: eligible studies included published randomized controlled trials (RCT) as well as retrospective cohort studies (RCS); 2) patients with hepatocellular carcinoma diagnosed by imaging or pathology and no chance of surgery; 3) treatment approaches: the experimental cohort received a combination remedy comprising HAIC, Lenvatinib, and a PD-1 inhibitor, whereas the control cohort was administered Lenvatinib in conjunction with a PD-1 inhibitor; 4) principal endpoints: the trial centered on evaluating ORR, DCR, OS, PFS, and the incidence of AEs, with the stipulation that at least one parameter related to survival was mandatory for assessment purposes.

Exclusion criteria: 1) inconsistent intervention measures; 2) observation of inconsistent outcome indicators; 3) articles were medical record reports, meeting abstracts, letters, a Meta-analysis, reviews, animal experiments, and repetitive articles; 4) no control group; 5) research that cannot obtain full text or data cannot be extracted.

### Data extraction and quality assessment

Data extraction from eligible studies was conducted independently by two reviewers, adhering to the predefined inclusion-exclusion criteria. To ensure accuracy, a third reviewer performed a cross-check of the extracted data. Any discrepancies that arose between the reviewers were resolved through consensus discussions. The data extracted encompassed several key variables: first author, treatment modalities, publication dates, sample sizes, participant demographics (including gender and age), Child-Pugh classification, ECOG PS, BCLC stage, and post-treatment outcome measures. These outcome measures included ORR, DCR, OS, and PFS. Additionally, AEs were recorded. It is noteworthy that all studies incorporated in this meta-analysis were retrospective. For assessing methodological quality, we employed the Newcastle Ottawa Scale (NOS) (13), providing a standardized framework for evaluating the studies’ rigor, with a total score of 9 points, including cohort selection, comparability, exposure, and ≥ 6 points were considered high quality.

### Statistical analysis

In this study, we employed RevMan 5.4 software to perform a systematic meta-analysis of the collected data. The hazard ratios (HRs), accompanied by their respective 95% confidence intervals (CIs), served as the primary effect measure for analyzing OS and PFS. For binary variables, including 1-and 2-year OS and PFS, ORR, DCR, and AEs, we employed relative risks (RR) and their corresponding 95% CIs as the effect indicators. Heterogeneity was assessed using the I^2^ statistic; a fixed-effects model was used when I^2^ was under 50% and the P-value was above 0.1, otherwise, we resorted to employing a random-effects model. Evaluating publication bias through the analysis of funnel plots. A P-value below 0.05 indicates significant disparities.

## Result

**Figure 1:**
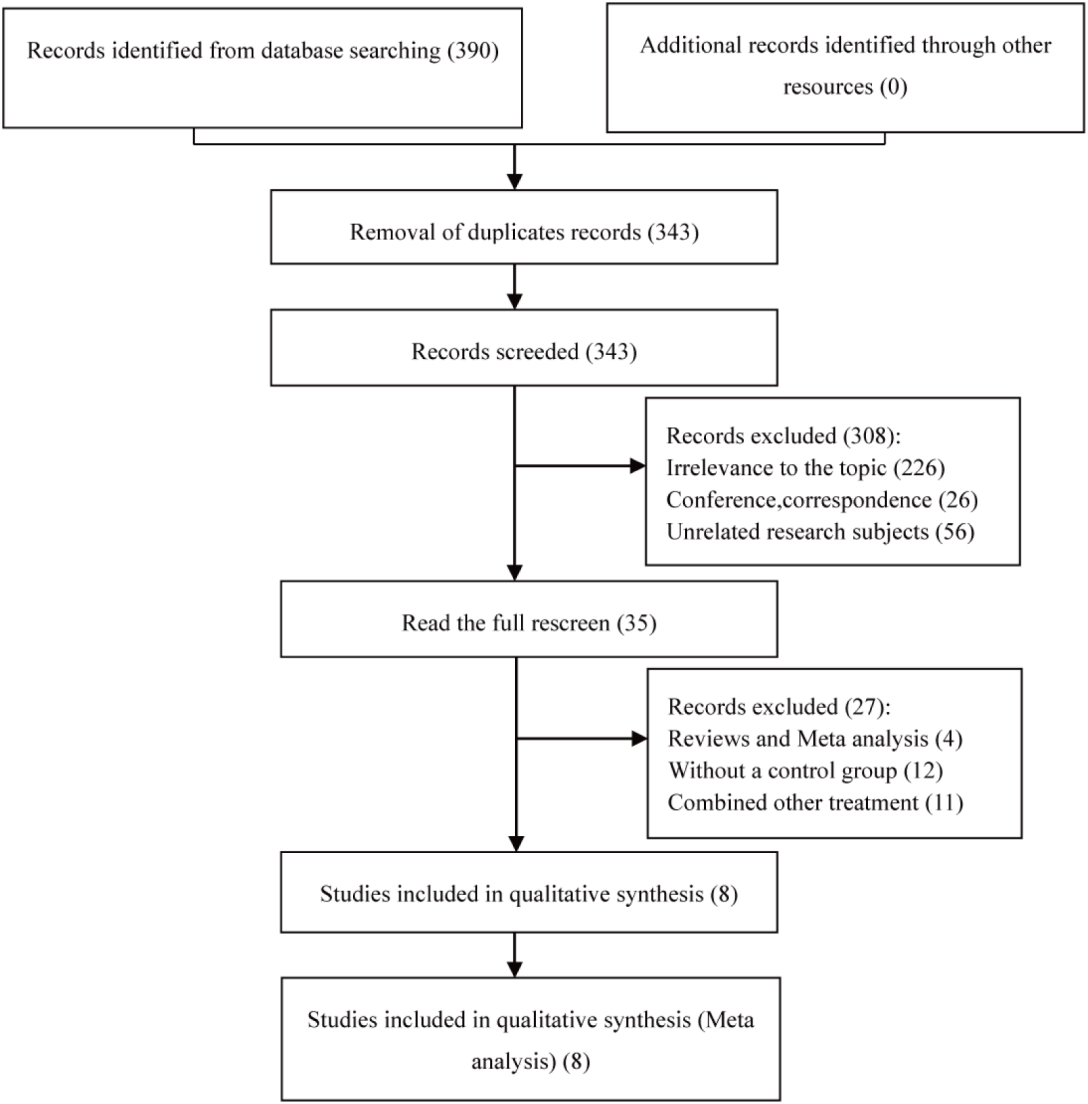
Literature screening and selection process.

Ultimately, the study encompassed 8 articles (14–21). A total of 390 eligible articles were retrieved, and 47 duplicates were automatically de-duplicated and manually excluded by EndNote; titles and abstracts were scrutinized to exclude off-topic articles, followed by a full-text review that culminated in the selection of 8 pertinent articles for this meta-analysis, all of which were retrospective cohort studies (RCS). The literature selection process is graphically represented in **Figure 1**.

### Study characteristics and risk of bias

The features of the incorporated studies are summarized in **Table 1**. A comprehensive analysis encompassing 1174 patients was conducted across 8 studies, with all participants hailing from China between the years 2021 to 2024. Specifically, 654 patients diagnosed with uHCC underwent HAIC-L-P triple therapy, whereas 520 patients received the L-P dual therapy. The NOS was applied to appraise the methodological rigor of the enrolled studies, with each study securing a score of 7 or higher on the NOS, indicating a high standard of quality, as shown in **Table 1**.

**Table 1:** Basic characteristics and quality evaluation.

Study	Country	Design	Group/Participants	Sex(M/F)	Age(years)	Child-Pugh class(A/B)	ECOG PS (0-1/2)	BLCL(B/C)	NOS score
Xu 2024 (14)	China	RCS	HAIC-L-P /103	91/12	52.0±8.82	84/19	96/7	4/99	7
			L-P /61	54/7	56.0±7.88	50/11	58/3	2/59	
Li rx 2024 (15)	China	RCS	HAIC-L-P /81	69/12	≥60:68 <60:13	/	/	9/109	8
			L-P /81	68/13	≥60:72 <60:9	/	/	13/101	
Diao lf 2023 (16)	China	RCS	HAIC-L-P /58	49/9	≤ 50:16 > 50:52	49/9	27/31	24/34	8
			L-P /63	50/13	≤ 50:5 > 50:57	55/8	23/40	25/38	
Guan rg 2024 (17)	China	RCS	HAIC-L-P /127	107/20	52.9±10.88	104/23	/	/	7
			L-P /103	94/9	54.01±11.54	84/19	/	/	
Chen 2021 (18)	China	RCS	HAIC-L-P /84	72/12	52±6.25	71/13	84/0	22 62	8
			L-P /86	71/15	53±6.5	75/11	86/0	21/65	
Fu yz 2023 (19)	China	RCS	HAIC-L-P /89	83/6	/	/	/	/	7
			L-P /53	50/3	/	/	/	/	
Mei 2021 (20)	China	RCS	HAIC-L-P /45	38/7	49.1±10.6	44/1	/	5/40	8
			L-P /25	18/7	49.1±10.6	22/3	/	3/22	
Lou yd 2024 (21)	China	RCS	HAIC-L-P /67	61/6	56.15 ± 7.78	56/11	/	/	8
			L-P /48	44/4	53.48 ± 10.39	37/11	/	/	

M, male; F, female; HAIC,Hepatic arterial infusion chemotherapy; L, lenvatinib; P, PD-1 inhibitors; ECOG, Eastern Cooperative Oncology Group; BCLC, Barcelona Clinic Liver Cancer.

NOS, the Newcastle–Ottawa Scale; RCS, retrospective cohort study; /, not reported.

### Meta-analysis outcomes

#### Response outcomes

**Figure 2:**
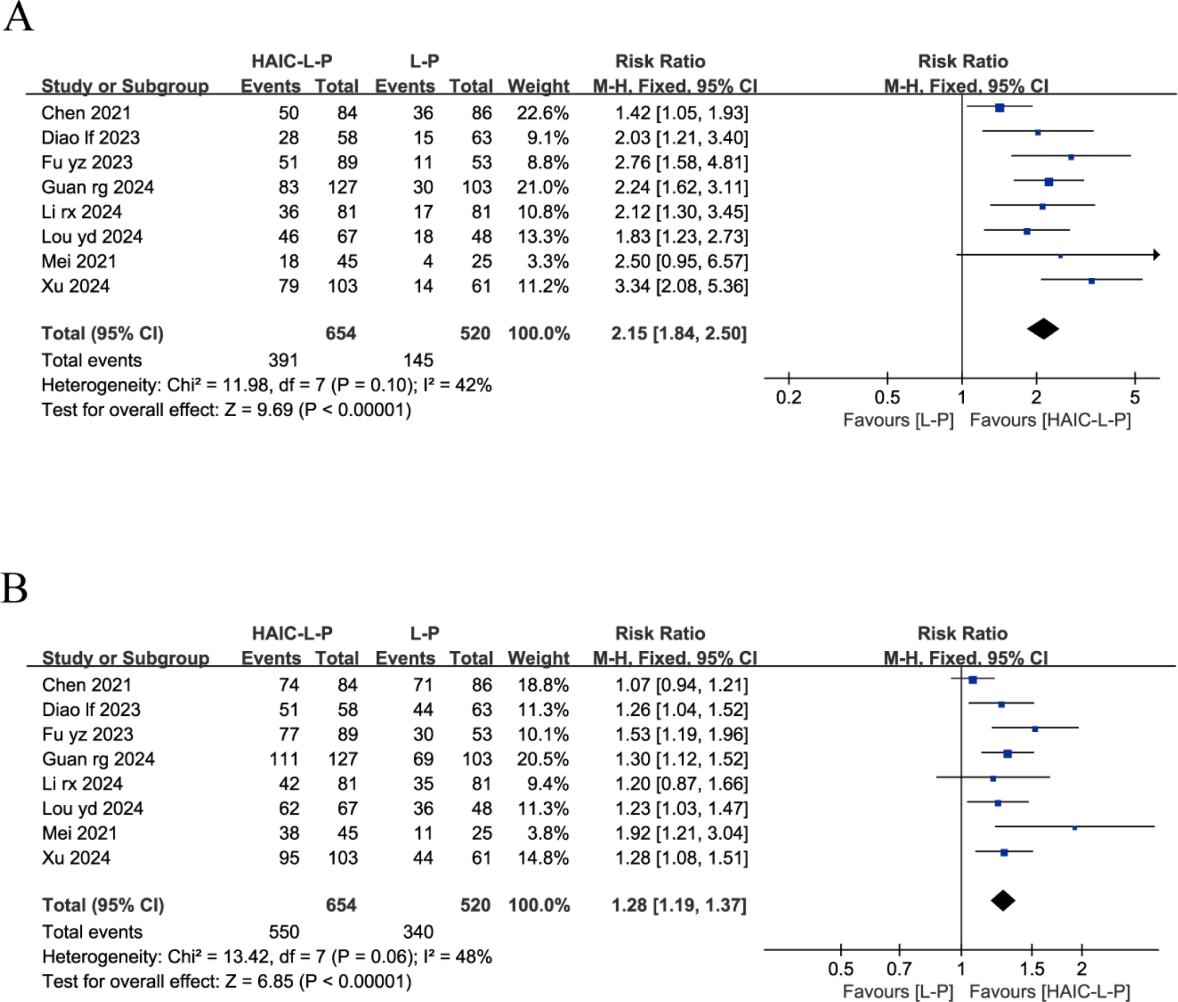
Fixed effect model of ORR **(A)** and DCR **(B)** for uHCC with HAIC-L-P vs L-P. ORR, objective response rate; DCR, disease control rate; uHCC, unresectable hepatocellular carcinoma; HAIC, Hepatic arterial infusion chemotherapy; L, lenvatinib; P, PD-1 inhibitors; CI, confidence intervals.

Eight studies reported on both the ORR and DCR across both groups. These studies exhibited no significant statistical heterogeneity, with I^2^ < 50%, prompting the use of a fixed-effects model for our analysis. The findings indicated that, compared to the L-P group, individuals with uHCC in the HAIC-L-P group experienced markedly improved ORR (RR = 2.15; 95% CI: 1.84–2.50; P < 0.00001) and DCR (RR = 1.28; 95% CI: 1.19–1.37; P < 0.00001), as depicted in **Figure 2**.

#### Overall survival and progression-free survival

**Figure 3:**
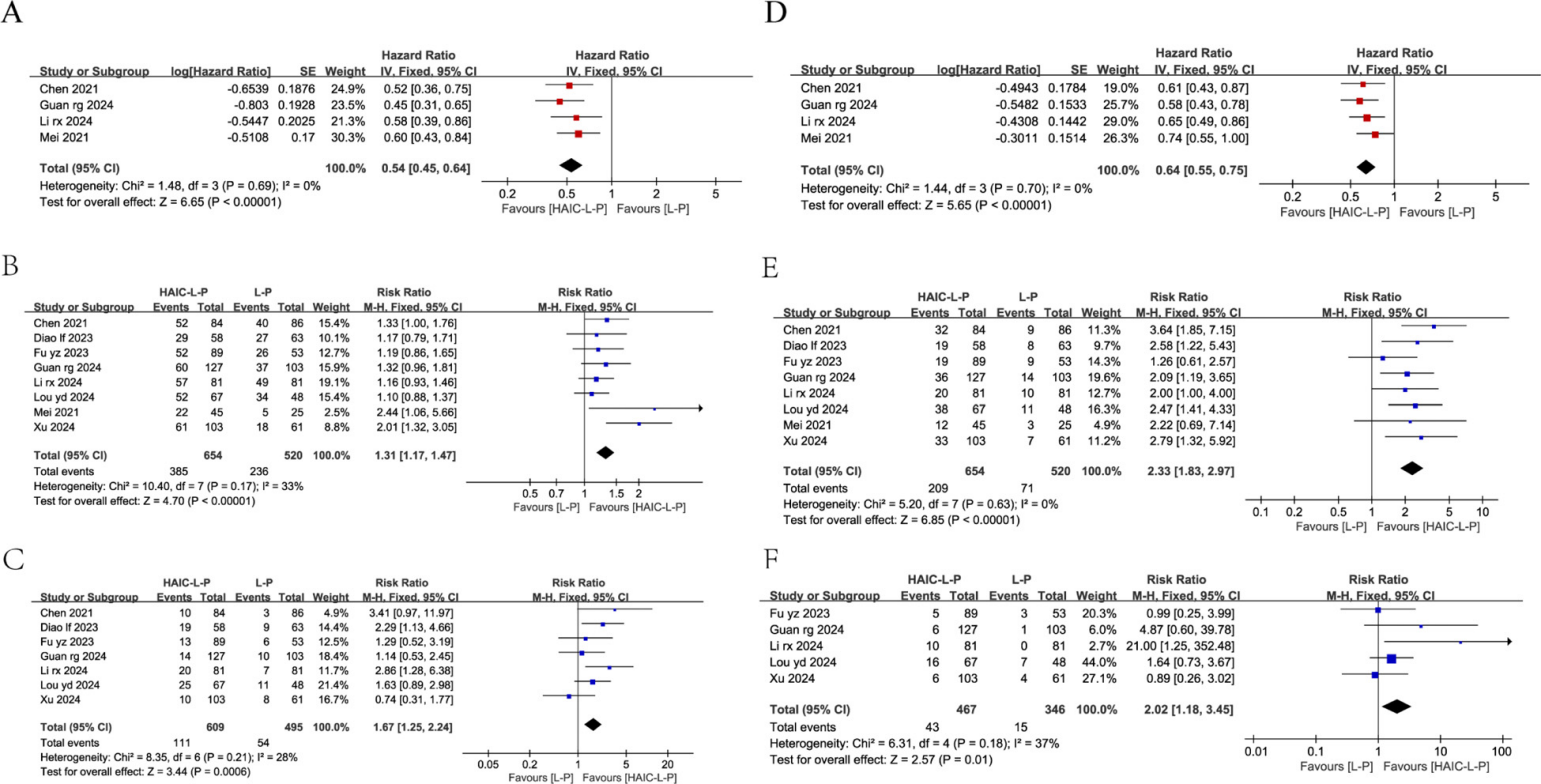
Fixed effect model of OS **(A)**, 1-year OS **(B)**, 2-year OS **(C)**, PFS **(D)**, 1-year PFS **(E)**, 2-year PFS **(F)** for uHCC with HAIC-L-P vs L-P. OS, overall survival; PFS, progression-free survival; uHCC, unresectable hepatocellular carcinoma; HAIC, Hepatic arterial infusion chemotherapy; L, lenvatinib; P, PD-1 inhibitors; CI, confidence intervals.

Only four studies reported OS and PFS data for both cohorts, including HRs and 95% CIs. Our analysis showed that the HAIC-L-P treatment notably improved OS (HR = 0.54, 95% CI: 0.45–0.64, P < 0.00001) and PFS (HR = 0.64, 95% CI: 0.55–0.75, P < 0.0001) in individuals with uHCC compared to the L-P regimen. The enhanced therapeutic benefit of HAIC-L-P is depicted in **Figure 3**.

Among the studies, eight reported 1-year OS and seven reported 2-year OS, while eight and five studies reported 1-year and 2-year PFS, respectively. Subsequent meta-analyses demonstrated that individuals with uHCC in the HAIC-L-P group demonstrated a markedly enhanced 1-year OS (RR = 1.31; 95% CI: 1.17–1.47, P < 0.00001), 2-year OS (RR = 1.67; 95% CI: 1.25–2.24, P = 0.0006), 1-year PFS (RR = 2.33; 95% CI: 1.83–2.97, P < 0.00001), and 2-year PFS (RR = 2.02; 95% CI: 1.18–3.45, P = 0.01) compared to the L-P group. The therapeutic benefit of the HAIC-L-P combination therapy was found to be superior to that of the L-P group, as depicted in **Figure 3**.

### Security assessment

#### Adverse events

All studies documented varying levels of AEs across both patient groups. Predominantly, the AEs were mild, and there were also reports of adverse reactions of grade ≥ 3, but there were no deaths. In terms of frequency of all grades AEs, the HAIC-L-P group exhibited a greater likelihood of experiencing Abdominal pain(RR =1.97, 95%CI: 1.27-3.08, P=0.003), Decreased appetite(RR =1.47, 95%CI: 1.21-1.78, P=0.0001), Vomiting(RR =2.60, 95%CI: 1.46-4.60, P=0.001), Elevated AST(RR =2.42, 95%CI: 1.41-4.17, P=0.001), Elevated ALT(RR =2.00, 95%CI: 1.34-2.98, P=0.0007), Thrombocytopenia(RR =2.00, 95%CI: 1.34-2.98, P=0.0007), Anaemia(RR =1.69, 95%CI: 1.23-2.32, P=0.001), Neutropenia(RR =2.80, 95%CI: 1.27-6.18, P=0.01), Hyperbilirubinacemia(RR =1.54, 95%CI: 1.03-2.29, P=0.03) than the L-P group.

Regarding AEs classified as grade 3 or above, the HAIC-L-P group exhibited elevated risk ratios for Vomiting (RR = 3.98, 95% CI: 1.51–10.50, P = 0.005), Elevated AST (RR = 2.67, 95% CI: 1.66–4.28, P = 0.0001), Elevated ALT (RR = 1.85, 95% CI: 1.20–2.84, P = 0.005), Thrombocytopenia (RR = 3.27, 95% CI: 1.81–5.91, P = 0.0001), Neutropenia (RR = 6.69, 95% CI: 2.08–21.52, P = 0.001), and Hyperbilirubinemia (RR = 2.78, 95% CI: 1.47–5.23, P = 0.001) compared to the L-P group, with these differences attaining statistical significance. A detailed overview of these findings is presented in **Table 2**.

**Table 2:** Adverse events of HAIC-L-P group vs L-P group.

Adverse Events	All grades			Grade 3-4		
	Studies	RR [95% CI]	P	Studies	RR [95% CI]	P
Diarrhea	8	0.86 [0.66, 1.12]	0.26	5	0.86 [0.37, 2.02]	0.73
Abdominal pain	6	1.97 [1.27, 3.08]	**0.003**	4	2.18 [0.90, 5.29]	0.08
Decreased appetite	7	1.47 [1.21, 1.78]	**0.0001**	3	2.15 [0.99, 4.67]	0.05
Hypertension	6	1.02 [0.87, 1.20]	0.76	4	1.03[0.58, 1.84]	0.92
Vomiting	6	2.60 [1.46, 4.60]	**0.001**	5	3.98[1.51, 10.50]	**0.005**
Rash	7	1.02 [0.75, 1.37]	0.92	4	0.18[0.49, 1.69]	0.77
Hand-foot syndrome	2	0.75 [0.21, 2.72]	0.66	2	2.11[0.61, 7.24]	0.24
Fatigue	8	1.19 [0.99, 1.43]	0.07	4	0.87[0.42, 1.81]	0.71
Elevated AST	5	2.42 [1.41, 4.17]	**0.001**	5	2.67[1.66, 4.28]	**0.0001**
Elevated ALT	7	2.00 [1.34, 2.98]	**0.0007**	6	1.85[1.20, 2.84]	**0.005**
Thrombocytopenia	7	2.63[1.24, 5.58]	**0.01**	6	3.27[1.81, 5.91]	**0.0001**
Hypothyroidism	8	1.06[0.81, 1.38]	0.67	4	1.50[0.66, 3.43]	0.33
Anaemia	4	1.69[1.23, 2.32]	**0.001**	3	2.73 [0.46, 16.02]	0.27
Neutropenia	6	2.80[1.27, 6.18]	**0.01**	4	6.69 [2.08, 21.52]	**0.001**
Proteinuria	5	1.26[0.90, 1.75]	0.18	4	1.41[0.58, 3.38]	0.45
Hyperbilirubinacemia	7	1.54[1.03, 2.29]	**0.03**	5	2.78[1.47, 5.23]	**0.002**

AST, aspartate aminotransferase; ALT, alanine aminotransferase; RR, relative risk; CI, confidence intervals. The bold values indicate that compared to the L-P group, the HAIC-L-P group of uHCC patients experienced more frequent adverse events, which should be taken seriously. The bold values indicate that compared to the L-P group, the HAIC-L-P group of uHCC patients experienced more frequent adverse events.

#### Publication bias

**Figure 4:**
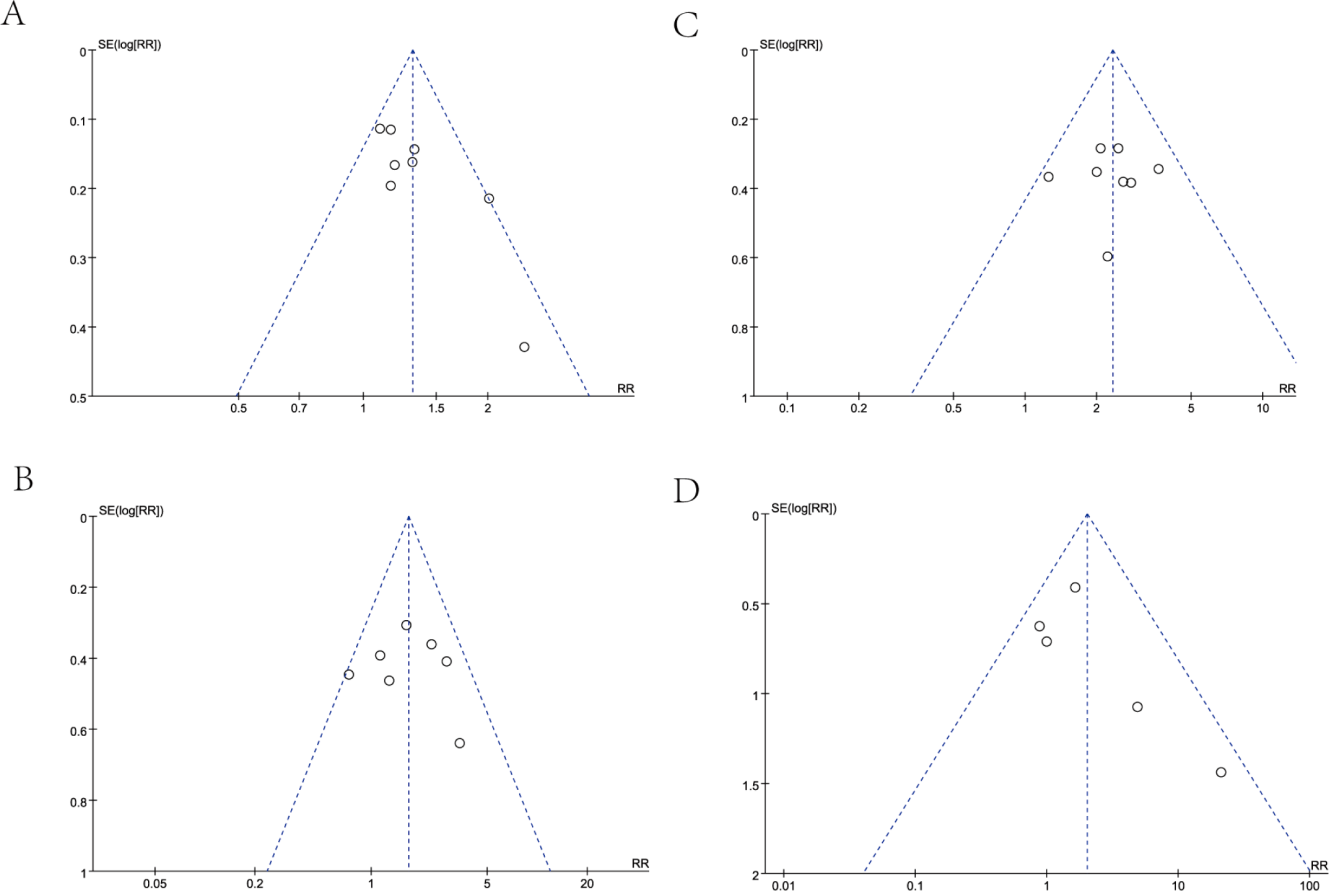
funnel plot of 1-year OS **(A)**, 2-year OS **(B)**, 1-year PFS **(C)**, and 2-year PFS **(D)**.

In our analysis, we employed a funnel plot to evaluate the long-term efficacy between the two treatment groups, specifically examining OS at 1-year and 2-year follow-ups, as well as PFS at the same time points. The results of each funnel plot demonstrated that the respective data points from the included studies were all encompassed within the funnel plot and displayed a generally symmetrical distribution (**Figure 4**). In general, the likelihood of publication bias is minimal, given the symmetrical distribution of scatter points within the inverted funnel plot.

## Discussion

Advanced HCC has a poor prognosis and limited treatment options. Sorafenib (22) recommended by guidelines as a first-line treatment, fails to provide durable antitumor effects, leading to drug resistance and tumor recurrence. With the maturation of interventional therapies, HAIC has gained widespread recognition for its efficacy and minimal systemic side effects due to its localized nature and high drug concentration in the liver (23). Previous research has indicated that HAIC has the potential to augment local drug concentrations, subsequently enhancing therapeutic effectiveness, when compared to transarterial chemoembolization (TACE), HAIC, in combination with oxaliplatin and 5-fluorouracil (5-FU), has demonstrated superior ORR and OS (24). HAIC-based combination treatments target the enhancement of survival times in individuals with uHCC. Hepatitis C virus (HCV) is unequivocally recognized as a principal etiology of chronic liver disease, cirrhosis, and HCC. HCV is classified into distinct genotypes and subtypes based on geographical distribution and transmission risk profiles. These genotypes and subtypes are intricately linked to viral load and are significant determinants of therapeutic efficacy and disease progression (25). In regions where HCV is endemic, HAIC emerges as a potential first-line treatment option, particularly for patients with advanced HCC characterized by microvascular invasion in the absence of extrahepatic metastasis. Moreover, the combination of local regional therapy with systemic targeted and immunotherapies has garnered significant attention in the treatment of uHCC, and a burgeoning corpus of experimental data validates the potency of the HAIC-lenvatinib-PD-1 axis in combating advanced HCC (26). Yet, a cohesive appraisal of this trimodal therapy’s efficacy and safety remains uncharted. Leveraging clinical trial evidence, our study delivers a holistic assessment of this integrated therapeutic approach for uHCC, thereby anchoring clinical practice in a robust scientific framework.

Our meta-analysis reveals that the HAIC-L-P group demonstrated superior outcomes over the L-P group, with extended OS and PFS, along with improved ORR and DCR. The heterogeneity among studies was minimal. We underscore the importance of scrutinizing the principal determinants of heterogeneity when interpreting clinical data and study methodologies. It is recommended that forthcoming research endeavors delve into these determinants to enhance the precision of treatment effect estimations. In this study’s indicators (ORR, DCR, OS, 1-year OS, 2-year OS, PFS, 1-year PFS, 2-year PFS), we assessed heterogeneity using I² (values <50%) indicating low heterogeneity, leading to the selection of the fixed-effects model. AEs associated with treatment were manageable and did not result in any mortalities. These results align with previous studies. While the L-P combination is an important treatment option for uHCC, its limitations in advanced disease highlight the need for enhanced therapeutic strategies. A recent investigation suggests the L-P group has demonstrated significant antitumor activity in patients with advanced uHCC, however, the HAIC-L-P triple therapy, employed as the initial treatment for HCC patients exhibiting macrovascular invasion, achieves superior ORR and DCR, furthermore, a greater number of patients became eligible for conversion surgery, and the adverse events were deemed tolerable (27). In addition, a recent clinical trial has demonstrated that the HAIC-L-P combination significantly enhances oncological responses and extends survival, thereby offering a more favorable survival prognosis for HCC patients who are unresponsive to TACE (28).

This improvement in patient prognosis with triple therapy is attributed to several mechanisms. On the one hand, HAIC delivers chemotherapeutic agents into the tumor-feeding arteries, inducing cytotoxic effects and creating an ischemic hypoxic environment that leads to tumor necrosis and promotes the production and release of tumor-specific antigens. Inhibitors of the PD-1 pathway may facilitate the generation of tumor antigen-specific memory T cells, thereby amplifying and perpetuating the patient’s immune response against the tumor (29), HAIC induces ischemic necrosis of tumor tissue while releasing a substantial amount of tumor-specific antigens, thereby enhancing the antitumor immune effects of PD-1 inhibitors (30). On the other hand, lenvatinib, a multi-kinase inhibitor known for its anti-angiogenic properties, can neutralize the vascularization triggered by hypoxia following HAIC. It normalizes blood vessels, fine-tunes the immune contexture of the tumor, and fosters the penetration of immune cells into the tumor mass, lenvatinib exhibits inhibitory effects on IFN-γ signal transduction in tumor cells through targeted modulation of FGFR, thereby amplifying the immunological effects of PD-1 inhibitors against HCC (31, 32). The combined use of PD-1 and lenvatinib can also reverse the immunosuppressive state within the tumor microenvironment, thereby increasing the immune response rate to PD-1 inhibitors (33). The deployment of the triple therapy can establish a robust positive feedback mechanism against tumors, engendering profound and enduring therapeutic responses through a multitude of synergistic pathways.

This study appraised the safety profile of the integrated therapeutic approach, including HAIC, for uHCC patient treatment. The findings indicate that combination therapy, while enhancing therapeutic efficacy, also results in the occurrence of inevitable adverse reactions. The HAIC-L-P group has a higher risk of grade≥3 AEs, including vomiting, bone marrow suppression (thrombocytopenia, neutropenia), and liver function damage (elevated AST, elevated ALT, hyperbilirubinemia). Therefore, special attention should be paid to repeated blood tests for routine, liver function, and if thrombocytopenia, neutropenia, and liver function abnormalities occur, timely treatment with recombinant human interleukin 11, granulocyte colony-stimulating factor injection, and hepatoprotective drugs should be administered. If necessary, the infusion of chemotherapeutic agents should be slowed down or paused. If long-term recurrent vomiting occurs after HAIC treatment, rehydration should be intensified to maintain water-electrolyte balance and intravenous nutritional support should be given appropriately. Other adverse reactions should also be vigilantly monitored for timely treatment. For uHCC patients, although the HAIC-L-P triple therapy may cause more adverse reactions, these can be managed by extending hospital stays and symptomatic treatment, indicating that these reactions are controllable. Consequently, in clinical settings, vigilant attention must be given to AEs induced by the therapeutic regimen to guarantee the safety of treatment administration.

This meta-analysis has some limitations. Firstly, it’s crucial to recognize that this meta-analysis, like all others in the field, grapples with inherent variability across the encompassed trials due to differences in baseline patient demographics, disease classification, and therapeutic approaches. Secondly, considering that this study is rooted in a retrospective cohort design and the small number of studies, which may introduce selection bias, publication bias, and heterogeneity concerns, our findings necessitate further validation through more prospective, randomized controlled trials. These trials will furnish us with more robust evidence to ascertain the efficacy of the HAIC-L-P regimen in the treatment of uHCC. This methodological shortfall could inadvertently lead to an overestimation or underestimation of the reported treatment effect sizes. Owing to the scarcity of original studies and available extractable data, this study could not perform further subgroup analyses. Given the Chinese origin of all published studies, the majority of HCC cases in China are primarily associated with hepatitis B virus infection, while in other regions, HCV is the main culprit. Due to variations in healthcare infrastructure, resource availability, and cultural practices, the treatment approaches for viral hepatitis differ across regions, potentially impacting the universality of our research findings, the applicability of these findings to Western populations might be subject to caveats. In our study, the benefits of HAIC-L-P triple therapy may be related to the etiology of the disease. Therefore, we recommend that future analyses should aim to include studies from diverse geographical areas to enhance the global applicability of the research results. By conducting or incorporating international studies in future meta-analyses, a more comprehensive understanding of the efficacy of HCC treatments across different populations can be achieved.

## Data Availability

The original contributions presented in the study are included in the article/supplementary material. Further inquiries can be directed to the corresponding author.
